# P-1753. Herpesvirus Seropositivity Associated with Increased Inflammation in Pediatric Sepsis

**DOI:** 10.1093/ofid/ofaf695.1924

**Published:** 2026-01-11

**Authors:** Zachary Aldewereld, Joe Carcillo

**Affiliations:** Univ of Pittsburgh / UPMC Children's Hospital of Pittsburgh, Pittsburgh, Pennsylvania; Univ of Pittsburgh / UPMC Children's Hospital of Pittsburgh, Pittsburgh, Pennsylvania

## Abstract

**Background:**

Sepsis remains a significant cause of pediatric mortality and morbidity. We recently reported that EBV seropositivity was independently associated with increased mortality in pediatric sepsis, and CMV and HSV seropositivity were associated with increased mortality in univariable analysis. This study aims to further understand the pathophysiological derangements associated with these findings.

**Figure:**
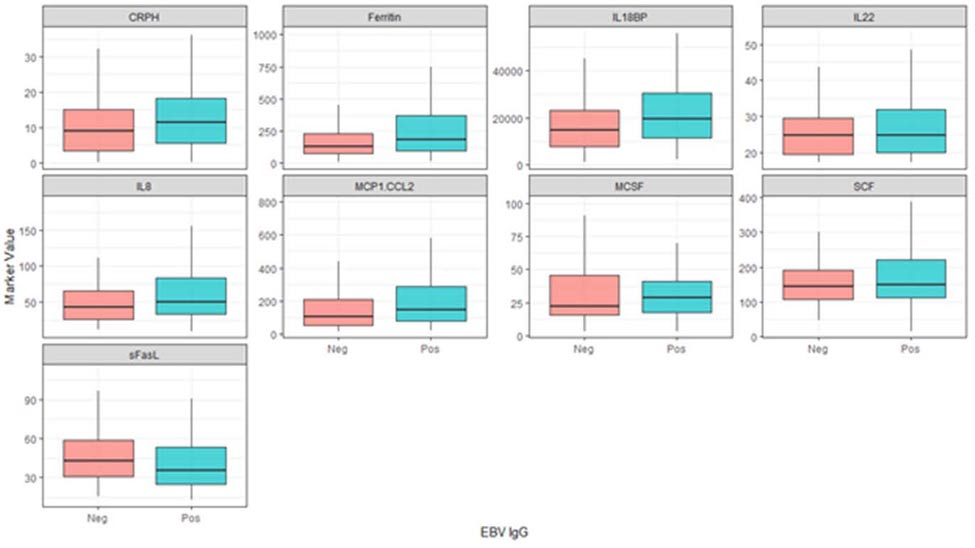
Comparison of biomarkers significantly different across EBV, CMV, and HSV serologies, divided by EBV serostatus Patients with EBV had significantly higher levels of CRP, Ferritin, and multiple cytokines. (outliers removed from plots)

**Figure:**
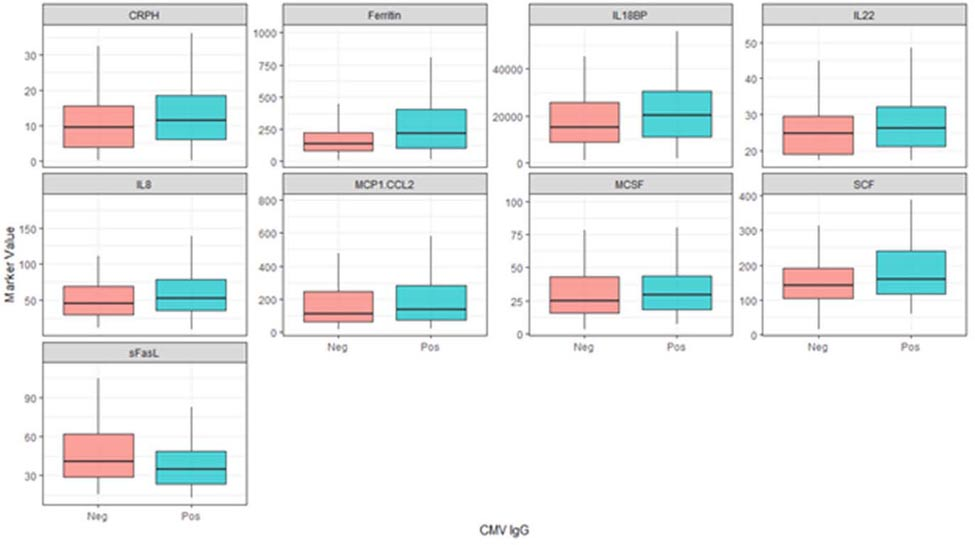
Comparison of biomarkers significantly different across EBV, CMV, and HSV serologies, divided by CMV serostatus Patients with CMV had significantly higher levels of CRP, Ferritin, and multiple cytokines. (outliers removed from plots)

**Methods:**

Secondary analysis of 401 pediatric sepsis patients from 9 Pediatric Intensive Care Units. Samples were tested by serology (IgG) for EBV, CMV, HSV, and HHV6. Inflammatory markers and cytokines/chemokines tested early in the course (first four days) were compared based on serologic status. Univariable associations were tested with Wilcoxon rank sum test.

**Figure:**
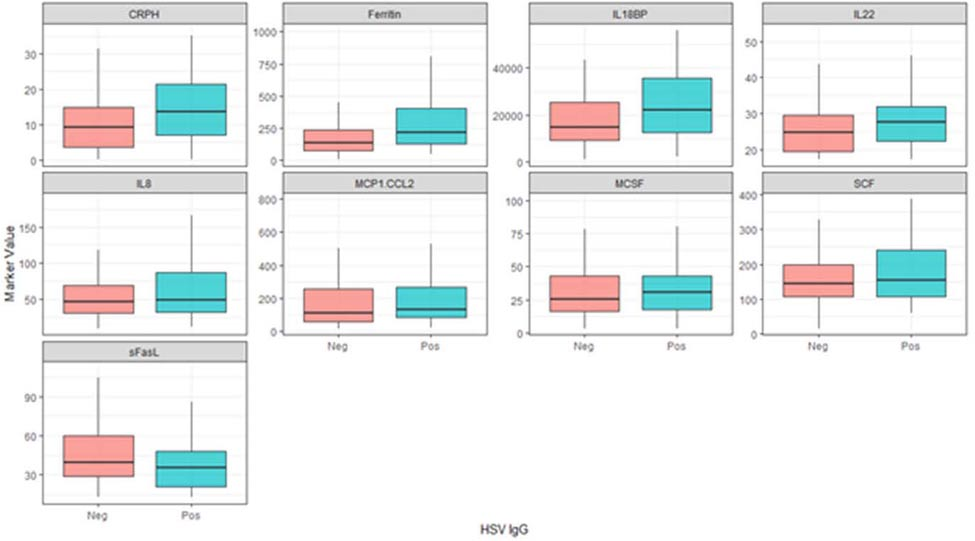
Comparison of biomarkers significantly different across EBV, CMV, and HSV serologies, divided by HSV serostatus Patients with HSV had significantly higher levels of CRP, Ferritin, and multiple cytokines. (outliers removed from plots)

**Results:**

Patients seropositive for EBV were more inflamed early in their course than seronegative patients (CRP 11.7 vs 9.1, p=0.008; Ferritin 263 vs 146, p< 0.001) with significantly higher levels of multiple cytokines/chemokines, specifically TNF (75 vs 67, p=0.044), IL6 (11 vs 8, p=0.038), IL8 (64 vs 44, p< 0.001), IL10 (24 vs 20, p=0.04), IL17A (20 vs 18.3, p=0.007), IL18BP (20358 vs 15280, p=0.004), IL22 (27 vs 25, p=0.049), CXCL9 (952 vs 727, p=0.029), MCP-1 (223 vs 108, p< 0.001), M-CSF (33 vs 23, p=0.01), and SCF (163 vs 143, p=0.028). Similar patterns were seen for CMV and HSV seropositivity.

**Conclusion:**

Seropositivity for herpesviruses is associated with greater inflammation at or near the time of presentation with sepsis. These findings suggest that herpesvirus latency may contribute to a greater inflammatory response in the setting of pediatric sepsis.

**Disclosures:**

All Authors: No reported disclosures

